# The role of cytoreductive nephrectomy in renal cell carcinoma patients with liver metastasis

**DOI:** 10.17305/bjbms.2020.4896

**Published:** 2021-04

**Authors:** Boda Guo, Shengjing Liu, Miao Wang, Huimin Hou, Ming Liu

**Affiliations:** 1 Department of Urology, Beijing Hospital, National Center of Gerontology; Institute of Geriatric Medicine, Chinese Academy of Medical Sciences, Beijing, China; 2 Graduate School of Peking Union Medical College, Beijing, China; 3 Department of Andrology, Xiyuan Hospital of China Academy of Chinese Medical Sciences, Beijing, China

**Keywords:** Cytoreductive nephrectomy, renal cell carcinoma, liver metastasis

## Abstract

It is widely accepted that renal cell carcinoma (RCC) with liver metastasis (LM) carries a dismal prognosis. We aimed to explore the value of cytoreductive nephrectomy among these patients. Patients were extracted from the Surveillance, Epidemiology, and End Results (SEER) database between 2010 and 2017. The univariate and multivariate Cox proportional hazards models were conducted to select the prognostic predictors of survival. Patients were divided into nephrectomy and non-nephrectomy groups. Propensity score-matching (PSM) analyses were applied to reduce the above factors’ differences between the groups. Overall survival (OS) was compared by Kaplan–Meier analyses. Data from 683 patients were extracted from the database. The univariate Cox regression and multivariate Cox regression revealed that factors including age, histologic type, T and N stages, lung metastasis, brain metastasis, and nephrectomy were significant predictors of survival in the patients. After the PSM analyses, we found that nephrectomy prolonged OS. Nephrectomy can prolong OS in eligible RCC patients with LM.

## INTRODUCTION

Renal cell carcinoma (RCC) has been acknowledged as a significant global health challenge with rising incidence and mortality. It is estimated that the number of newly diagnosed RCC cases and cancer-caused deaths from RCC in 2018 were 403,262 and 175,098, respectively [1]. While a growing number of patients with early-stage RCC are diagnosed using modern imaging techniques, 25–30% of RCC patients present with distant metastasis at initial diagnosis [2]. Liver is one of the most common metastatic sites of RCC, presenting in 23.6% of cases of newly diagnosed metastatic RCC (mRCC) [3].

The optimal treatment strategy for mRCC has not been established yet [4]. Comprehensive treatment including surgery, targeted therapy, and immunotherapy has provided more options for mRCC patients, which brings a significant survival benefit [5-11]. The role of cytoreductive nephrectomy (CN) has been debated without resolution. CN was considered as a viable therapeutic strategy for RCC patients with liver metastasis (LM) in some studies [12-16]. By contrast, some literature showed RCC patients with LM might not be suitable candidates for CN. According to the National Comprehensive Cancer Network (NCCN) clinical practice guidelines of kidney cancer (version 2.2020), CN is recommended for patients diagnosed with primary RCC and distant metastasis including the lung, bone, and brain but not the liver [17]. In addition, a previous study regarded LM as a contraindication to CN [18]. Since LM from RCC usually portends a poor prognosis [19] and is a predictor of widespread metastases [20], CN is seldom performed in this setting.

The Surveillance, Epidemiology, and End Results (SEER) database was built by the American National Cancer Institute (NCI), covering approximately 34.6% of the U.S. population. This representative database is suitable to correct problems inherent in single-center studies that use small sample sizes. In this study, we aim to explore the value of CN on prognosis of renal cell carcinoma with liver metastasis (RCCLM) patients.

## MATERIALS AND METHODS

### Source of data

Patient data were retrieved from the SEER database between 2010 and 2017, since the sites of metastases in RCC patients were not recorded until 2010 and the latest date available was 2017. Data extracted for each record include age, gender, race, histologic type, laterality, T stage, N stage, grade, synchronous distant metastatic sites involving bone, brain, liver and lung, surgery of primary site, survival time, the reason why surgery was not performed, vital status, and cause-specific death classification. The SEER*Stat software version 8.3.6 (NCI, US; https://seer.cancer.gov/seerstat/) was utilized to achieve this. Histologic types were limited to the following four variants according to the International Classification of Diseases for Oncology, 3rd edition (ICD-O-3) standard: (I) clear cell (ccRCC) [8310/3: clear-cell adenocarcinoma, not otherwise specified (NOS); 8322/3: water clear-cell adenocarcinoma; 8313/3: clear-cell adenocarcinoma]; (II) papillary (pRCC) [8260/3: papillary adenocarcinoma, NOS]; (III) chromophobe (chRCC) [8317/3: RCC, chromophobe type; 8270/3: chromophobe carcinoma]; (IV) collecting duct (CDC) [8319/3: collecting duct carcinoma]. The primary endpoint of this study was overall survival (OS), which was calculated from the date of diagnosis to the date of death from any cause.

**FIGURE 1:**
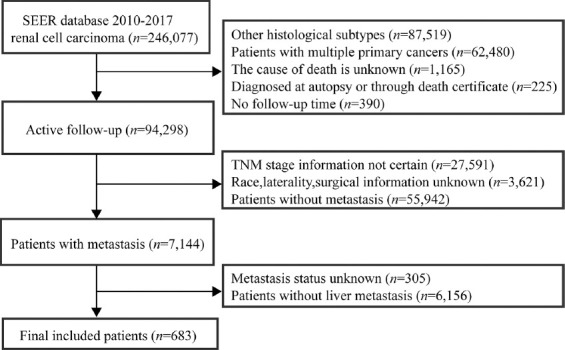
The flowchart of data screening. SEER: Surveillance, Epidemiology, and End Results; TNM: Tumor-node-metastasis.

The inclusion and exclusion criteria are summarized in Figure 1. The inclusion criteria were defined as: 1) histologically confirmed RCC with a primary site labeled as “C64.9 Kidney, NOS”; 2) complete follow-up information; 3) complete tumor-node-metastasis (TNM) classification data; and 4) complete partial nephrectomy or radical nephrectomy information (site-specific surgery codes: 30, 40, 50, 70, and 80).

The exclusion criteria in this study were as follows: 1) tumor was diagnosed solely on autopsy or death certificate or source information unknown; 2) missing or incomplete survival time (i.e., survival at 0 day); 3) multiple primary cancers; and 4) unknown race and tumor metastatic location.

### Ethical statement

Since the data released from the SEER database did not include any private information that could identify the patients, obtaining approval of this study by the institutional review board was not required.

### Statistical analysis

Baseline characteristics between different cohorts were compared using the Chi-squared test or Fisher’s exact test for the categorical variables. All data were obtained using SEER*Stat Software version 8.3.6. As a continuous variable, age was categorically divided based on the optimal cut-off value generated by X-tile software version 3.6.1 (Yale University School of Medicine, US). The univariate and multivariate Cox proportional hazards models were used to find the variables that may affect prognosis. All variables with a *p* value <0.1 in the univariate analysis were entered into the multivariate analysis. To reduce the heterogeneity of the clinicopathological characteristics between the groups, propensity score-matching (PSM) analyses at a 1:1 ratio by using the “nearest neighbor” method was applied to eliminate the effect of selection bias between the groups according to prognostic factors revealed from the multivariate analysis. The survival analyses before and after PSM were estimated by Kaplan–Meier (K–M) method. Two-tailed values of *p* < 0.05 in the multivariate analysis were considered significant. All statistical analyses were performed using R software version 3.6.1 [21].

## RESULTS

### Baseline characteristics and prognostic factors

A total of 683 eligible patients from 2010 to 2017 were identified from the SEER database (Table 1). The median age of all patients was 62 years, while the majority of cases were male and white (67.9% and 81.0%, respectively) and the median OS was 6 months. Based on the optimal cut-off value in age (age <55, age 55–73, and age >73), nearly a quarter of RCCLM patients (24.6%) were under 55 years old. The most common histologic type was ccRCC (85.8%), followed by pRCC (10.1%) and chRCC (2.6%). RCCLM patients were more likely to have additional lung metastasis (66.2%), but not brain (11.1%) or bone (34.1%) metastasis. Interestingly, in patients with RCCLM, there were more cases of T3 stage (44.1%), compared with T4 stage (23.6%). In addition, a minority of patients (43.0%) underwent surgery. Among 389 patients who did not undergo CN, 371 (95.4%) patients were not recommended for CN. Other reasons for not undergoing CN include that the death of patient happens before the recommended surgery, etc. Factors that were statistically associated with OS in the univariate analysis were age, histologic type, laterality, T stage, N stage, grade, lung metastasis, brain metastasis, bone metastasis, and nephrectomy. A multivariate analysis was carried out on the above statistically significant factors. Finally, age, histologic type, T stage, N stage, lung metastasis, brain metastasis, and nephrectomy were revealed as significant independent predictors of OS.

**TABLE 1:**
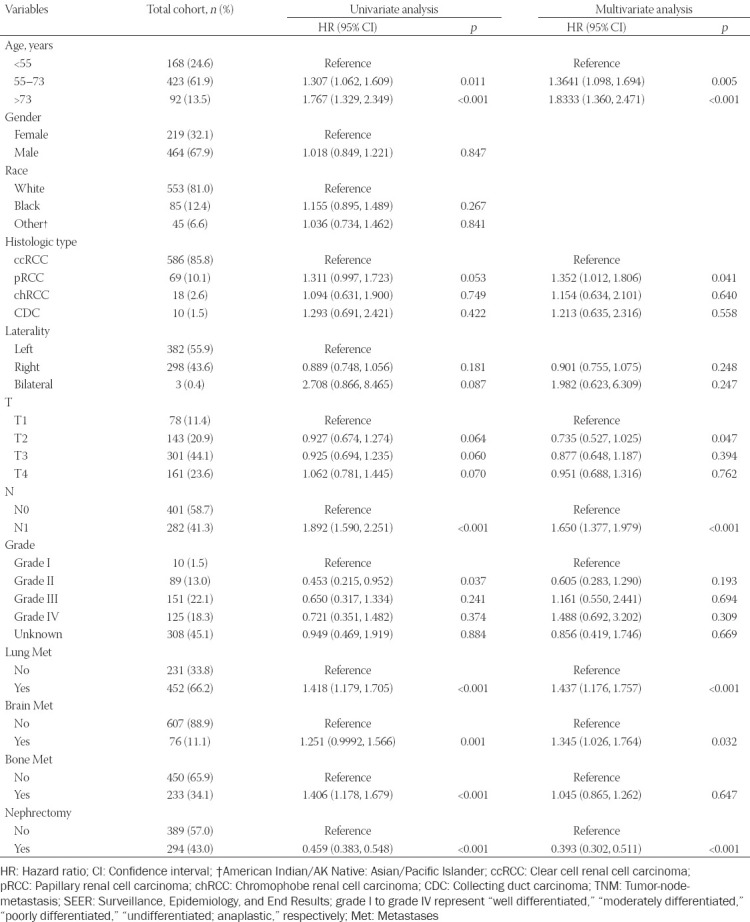
Patient characteristics of the total cohort and Cox regression analyses in the training cohort

### PSM analyses

**FIGURE 2:**
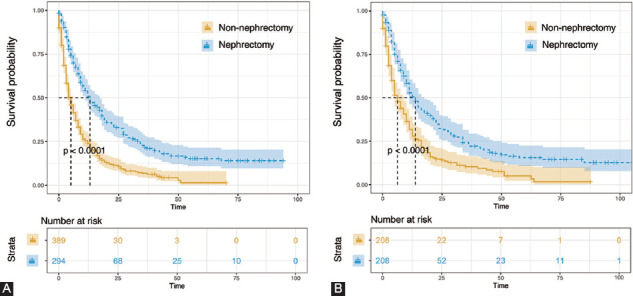
Kaplan–Meier analyses for cancer-specific survival in patients with or without nephrectomy before matching (A) and after matching (B).

After PSM, 208 patients undergoing CN matched 208 patients that did not undergo surgery. A caliper width of 0.005 was adopted. Basic characteristics were well balanced across all confounding factors (Table 2). Whether before or after PSM, the OS of patients who underwent nephrectomy was significantly longer than those without surgery (median OS before PSM: 9 months vs. 4 months, *p* < 0.0001, Figure 2A; and median OS after PSM: 9 months vs. 4 months, *p* < 0.0001, Figure 2B, respectively).

**TABLE 2:**
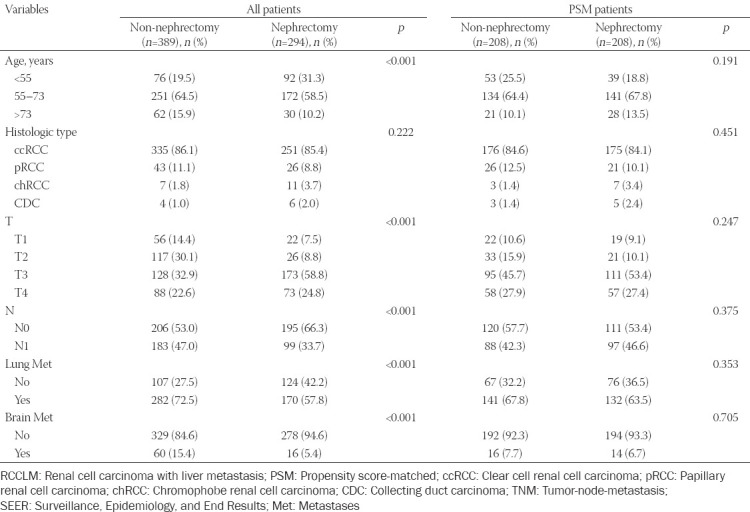
Characteristics of all RCCLM patients and propensity score-matching analysis for nephrectomy and non-nephrectomy groups

## DISCUSSION

Therapy options for metastatic RCC have evolved significantly over the past decade [22-25]. However, there is no consensus on the optimal clinical strategy to treat RCCLM [26]. As mentioned in the introduction section, surgery at the primary site is not recommended for RCCLM by the NCCN guideline [17] and a study from the U.S. [18]. The potential reasons are as follows. First, this American study evaluated patients who received interleukin-2, making its utility in the current targeted therapy era unknown. Second, LM is one of the negative predictors of survival in patients who undergo CN [27]. Third, LM is associated with the early postoperative complications of CN and prolonged length of stay [28]. In addition, evidence supporting the role of CN in RCCLM patients comes from two small-scale case-control studies [12,13] and case reports [14-16], which may lack persuasion. The shortage of relevant research evidence also makes it difficult in selecting candidates for CN in RCCLM. Although more than half of the patients (54.3%) in our study were not recommended for CN, it is noteworthy that CN can bring significant survival benefits in eligible patients as shown in our study. There is no doubt that patient selection in our study likely drives the survival outcomes. However, CN appears to be a feasible therapy in carefully selected RCCLM patients.

Prognostic factors for OS of RCCLM were identified in our study. Hence, tailored clinical interventions can be planned based on these factors. Interestingly, we found that nearly a quarter of RCCLM patients were under 55 years old and that the proportion of RCCLM patients with N1 stage was less than those with N0. These findings should be treated with reservation because of the relative lack of patients in each subgroup, and further studies should be conducted to confirm our results. Our data suggest that patients younger than 55 years old and who present with N0 stage also need close follow-up.

There are several limitations in our study. Firstly, we do not have information regarding patient performance status, systemic therapy given, or local treatment status of LM. Secondly, due to the limitations of the SEER database, information such as details of the surgery and distant organ metastasis could not be obtained, which hindered further prognostic analyses.

## CONCLUSION

To the best of our knowledge, this is the largest study to date exploring the role of CN in RCCLM. We conclude that CN represents a valid option for well-selected patients with RCCLM.
